# Efficacy and safety of Xiyanping for COVID-2019: A protocol for systematic review and meta-analysis

**DOI:** 10.1097/MD.0000000000022962

**Published:** 2020-11-13

**Authors:** Hui Zhou, Dongqiong Chen, Yuan Zhang, Qianru Zhu, Yan Yang, Yan Liu, Guangming Gong, Chunguang Xie

**Affiliations:** Hospital of Chengdu University of Traditional Chinese Medicine, Chengdu, Sichuan Province, P.R. China.

**Keywords:** coronavirus disease 2019, protocol, systematic review and meta-analysis, Xiyanping

## Abstract

**Background:**

Coronavirus disease 2019 (COVID-19) is a global pandemic caused by the severe acute respiratory syndrome coronavirus-2.COVID-19 is highly pathogenic and infectious. COVID-19 epidemic is still spreading all over the world, and there is no sign of stopping at present. There is no specific cure for this disease, and the clinical management mainly depends on supportive treatment. Xiyanping is widely used in treating COVID-19 in China. However, there is no evidence that Xiyanping is effective and safe for COVID-19.

**Methods:**

A comprehensive literature search will be conducted. Two methodological trained researchers will read the title, abstract, and full texts and independently select the qualified literature according to inclusion and exclusion criteria. After assessment of the risk of bias and data extraction, we will conduct meta-analysis for outcomes related to COVID-19. The heterogeneity of data will be investigated by Cochrane *X*^*2*^ and *I*^*2*^ tests. Then publication bias assessment will be conducted by funnel plot analysis and Egger test.

**Results:**

The results of our research will be published in a peer-reviewed journal.

**Conclusion:**

Our study aims to systematically present the clinical evidence of Xiyanping in the treatment of COVID-19, which will be of guiding significance for further research and clinical practice.

**Open Science Framework registration number:**

10.17605/OFS.IO/SW75F.

## 1 Introduction

Coronavirus disease 2019 (COVID-19) is a global pandemic caused by the severe acute respiratory syndrome coronavirus-2 (SARS-CoV-2).^[1]^ COVID-19 is highly pathogenic and infectious.^[2]^ Johns Hopkins University released the latest statistics on the epidemic situation of COVID-19. As of 9:28 on August 22, 2020, 22.98 million people had been infected and more than 800,000 deaths were reported worldwide.^[3]^ However, the COVID-19 epidemic is still spreading around the world, and there is no sign of stopping at present now vaccines have become the hope of human beings to overcome the epidemic situation. There are 167 vaccine projects in the world under study. However, even if the vaccine is successfully developed, it will not be able to end the COVID-19 pandemic itself. Therefore, vaccine is an important option for the treatment of COVID-19, but vaccine alone is far from enough, effective drugs are still necessary in clinic.

Due to the long development cycle and difficulty of new drugs, mining the existing drug therapy COVID-19 has become the focus of clinical research.^[4,5]^ In China, Chinese medicine has achieved satisfactory results in the prevention and treatment of COVID-19 and SARS. Among these traditional Chinese medicine preparations, Xiyanping is effective in the treatment of viral pneumonia.^[6–8]^ In 2018, 2 SARS-like coronaviruses were collected in Zhoushan, China, namely bat-SL-CoVZC45 and bat-SL-CoVZXC21. SARS-CoV-2 has a homology of 88% with bat-SL-CoVZC45 and bat-SL-CoVZXC21.^[9]^ Therefore, we boldly speculate that Xiyanping is equally effective in the treatment of COVID-19. The Seventh Edition of the “New Coronavirus Pneumonia Diagnosis and Treatment Plan (Trial)” issued by the National Health Commission recommends the Chinese medicine injection Xiyanping as one of the critical treatment drugs for COVID-19.^[10]^ There have been a study passed 3 clinical cases have shown that,^[11]^ Xiyanping has achieved satisfactory results in the treatment of severe new coronavirus pneumonia. However, these studies are conducted in local areas and cannot provide conclusive evidence to prove the safety and effectiveness of Xiyanping treatment.^[12]^ In this study, we aimed to summarize the latest evidence of Xiyanping in the treatment of COVID-19 through systematic review and meta-analysis. This study is necessary before further large-scale clinical studies are carried out. For clinicians, this study can provide some guidance for clinical practice.

## 2 Methods and analysis

### 2.1 Study registration

This study has been registered at Open Science Framework ( https://osf.io/) with a registration DOI: 10.17605/OFS.IO/SW75F. This systematic review protocol is reported in accordance with the preferred reporting items for systematic reviews and meta-analysis protocols checklist.^[13]^

### 2.2 Inclusion and exclusion criteria

#### 2.2.1 Study design

Randomized controlled trials (RCTs) can provide evidence about efficacy of intervention, so they will be included in this systematic review. However, the outbreak of COVID-19 is an urgent public health event, and it is difficult to carry out RCTs, so non-randomized controlled trials will also be included in this study, although non-randomized controlled trials (non-RCTs) may be more biased than RCTs.

#### 2.2.2 Participants

Participants with a laboratory-confirmed COVID-19 diagnosis will be included in this study. There will be no limitation about kits and detection methods. Also, there will be no restriction about age, sex, and severity of disease of participants.

#### 2.2.3 Intervention

Xiyanping in intervention group will be included. There will be no restrictions on the types, dosage forms, doses, and methods of use of Xiyanping.

#### 2.2.4 Outcomes

Since there are no core outcome sets for COVID-19, it is difficult to predefined what outcomes will be included in our study. In general, any outcome that can reflect the condition will be included in this study.

### 2.3 Study search

Three English database including PubMed, Embase, Cochrane Library Central Register of Controlled Trials, and 4 Chinese databases including China National Knowledge Infrastructure database, the VIP information resource integration service platform, Wanfang database,China Biology Medicine Disc (CBM) will be searched from its inception to September 1, 2020, without language limitation. Preprinted website including arXiv (http://arxiv.org/), BioRxiv (https://www.biorxiv.org/), F1000 (https://f1000.com/), and PeerJ Preprints (https://peerj.com/preprints/) will also be searched to find out more unpublished papers. In addition, Chinese Clinical Trial Registry (ChiCTR) and ClinicalTrials.gov will also be searched to find out ongoing research. A search strategy of the combination of controlled vocabulary and text words will be adopted. This work will be conducted by 2 authors (Hui Zhou and Dongqiong Chen) independently. We will simply present the search process of the cochrane library (Table 1). Adjusting different search methods according to different Chinese and English databases.

**Table 1:** Example of Cochrane search strategy.

Number	Search terms
1	Mesh descriptor:(Xiyanping injection) explode all trees
2	((Xiyanping injection^∗^) or (Xiyanping injection^∗^)or (Xiyanping injections^∗^) or (Xiyanping injections^∗^)):ti, ab, kw
3	Or 1–2
4	Mesh descriptor:(COVD-19) explode all trees
5	((2019 novel coronavirus infection^∗^)or (2019-nCoV infection^∗^) or (COVID-19 pandemic^∗^) or (coronavirus disease-19^∗^) or (2019-nCoV disease^∗^) or (COVID19^∗^) or (2019 novel coronavirus disease^∗^) or (coronavirus disease 2019^∗^))
6	Or 4–5
7	3 and 6

### 2.4 Study selection

**Figure 1:**
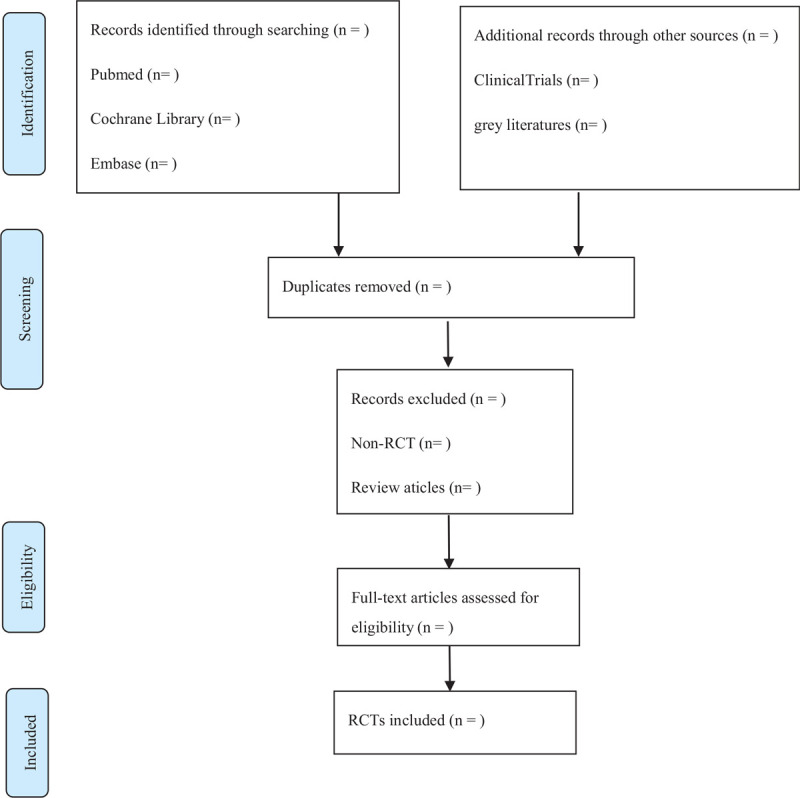
Flow chart of the study selection.

EndNote X9 will be used by 2 researchers (Hui Zhou and Dongqiong Chen) to screen the citations independently according to the predefined inclusion and exclusion criteria. Discrepancies between 2 authors will be solved by discussion with a third author (Chunguang Xie). A research flow chart will be drawn to show the whole process of research selection (Fig. 1).

### 2.5 Data extraction

Data extraction will be conducted by 2 independent authors (Hui Zhou and Dongqiong Chen) according to a prespecified form and checked by a third author (Chunguang Xie). The following data will be extracted: the first author's name, publication time, country, article title, article type, interventions in experimental and control group, course of treatment, severity of disease, number of patients in each group, ages and sex of patients, outcomes and adverse effect.

### 2.6 Risk of bias assessment

All the included studies will be evaluated based on the guidelines of Cochrane Handbook for Systematic Reviews of Interventions.^[14,15]^ Different risk of bias assessment tools will be used according to different types of research. The risk of bias of RCTs will be conducted using version 2 of the Cochrane risk-of-bias tool for randomized trials. The risk of bias In non-randomized studies of interventions tool will be used to assess the risk of bias of non-RCTs according to Cochrane Handbook.

### 2.7 Data analysis

Data analysis will be conducted using Stata 14.0 software. The effect measure of binary variable will be expressed as risk ratio or odds ratio (OR) and 95% confidence interval. For continuous variables, 95% confidence interval and mean difference or standardized mean difference (SMD) will be used. The number needed to treat will be calculated for the interpretation of results. Cochrane *X*^*2*^ and *I*^*2*^ tests will be conducted to assess the heterogeneity analysis between studies.When *P* < 50% and *I*^*2*^≥50%, a random effect model will be used. When *P* > 50% and *I*^*2*^ < 50%, then a fixed effect model will be used to calculate the effect size. The results of RCTs and non-RCTs will be analyzed and presented independently. Subgroup analysis will be conducted to explore the subgroup effects and investigate the source of heterogeneity. If there is a substantial heterogeneity and quantitative synthesis is not appropriate, the results will be presented in the form of tables and figures.

Publication bias and small-study effects will be evaluated by funnel plot and statistically investigated by Egger test^[16]^ with a *P* value boundary of 50%.

### 2.8 Ethics and dissemination

Meta-analysis is an analysis of previous research data and does not require ethical approval. The results of this study will be published in peer-reviewed journals.

## 3 Discussion

The purpose of this study is to summarize the efficacy of Xiyanping on COVID-19 and provide accurate guidance for further research and clinical application. Due to the difficulties in conducting clinical trials, this study will include randomized and non-randomized studies in order to collect clinical evidence as comprehensively as possible. Non-randomized research has more bias and confusion than randomized research, but it can provide evidence more conveniently. So we need to be more careful when interpreting non-RCT results. When assessing the risk of deviation, we will use the latest version of the tools recommended in the manual, which will ensure the correctness of our research from the methodological point of view. If any modification is required, we will update our protocol to include any changes in the entire research process.

## Author contributions

**Conceptualization:** Hui Zhou.

**Data curation:** Dongqiong Chen, Yuan Zhang, Qianru Zhu.

**Formal analysis:** Yan Yang, Yan Liu, Guangming Gong.

**Methodology:** Yan Yang, Yan Liu, Guangming Gong.

**Project administration:** Chunguang Xie.

**Resources:** Hui Zhou, Chunguang Xie.

**Software:** Hui Zhou, Chunguang Xie.

**Supervision:** Chunguang Xie.

**Writing – original draft:** Hui Zhou.

**Writing – review & editing:** Chunguang Xie.
